# Cereals as a Source of Bioactive Compounds with Anti-Hypertensive Activity and Their Intake in Times of COVID-19

**DOI:** 10.3390/foods11203231

**Published:** 2022-10-16

**Authors:** Abigail García-Castro, Alma Delia Román-Gutiérrez, Araceli Castañeda-Ovando, Raquel Cariño-Cortés, Otilio Arturo Acevedo-Sandoval, Patricia López-Perea, Fabiola Araceli Guzmán-Ortiz

**Affiliations:** 1 Área Académica de Química, Universidad Autónoma del Estado de Hidalgo, Carretera Pachuca–Tulancingo, Km 4.5 s/n, Mineral de la Reforma, Hidalgo 42184, Mexico; 2 Área Académica de Medicina, Instituto de Ciencias de la Salud, Universidad Autónoma del Estado de Hidalgo, Elíseo Ramírez Ulloa, 400, Doctores, Pachuca de Soto 42090, Mexico; 3 Área de Ingeniería Agroindustrial, Universidad Politécnica Francisco I. Madero, Francisco I. Madero, Hidalgo 42660, Mexico; 4 CONACYT, Universidad Autónoma del Estado de Hidalgo, Carretera Pachuca-Tulancingo Km 4.5 s/n, Mineral de la Reforma, Hidalgo 42184, Mexico

**Keywords:** cereals, COVID-19, diet therapy, drug therapy, hypertension, phytochemicals

## Abstract

Cereals have phytochemical compounds that can diminish the incidence of chronic diseases such as hypertension. The angiotensin-converting enzyme 2 (ACE2) participates in the modulation of blood pressure and is the principal receptor of the virus SARS-CoV-2. The inhibitors of the angiotensin-converting enzyme (ACE) and the block receptors of angiotensin II regulate the expression of ACE2; thus, they could be useful in the treatment of patients infected with SARS-CoV-2. The inferior peptides from 1 to 3 kDa and the hydrophobic amino acids are the best candidates to inhibit ACE, and these compounds are present in rice, corn, wheat, oats, sorghum, and barley. In addition, the vitamins C and E, phenolic acids, and flavonoids present in cereals show a reduction in the oxidative stress involved in the pathogenesis of hypertension. The influence of ACE on hypertension and COVID-19 has turned into a primary point of control and treatment from the nutritional perspective. The objective of this work was to describe the inhibitory effect of the angiotensin-converting enzyme that the bioactive compounds present in cereals possess in order to lower blood pressure and how their consumption could be associated with reducing the virulence of COVID-19.

## 1. Introduction

Cereals constitute an important part of the daily diet due to their high content of proteins, dietary fiber, and bioactive compounds with antioxidant and anti-inflammatory activities, which help prevent diseases related to metabolic syndromes such as obesity, cardiovascular diseases, and type 2 diabetes [1,2]. Wheat, oats, barley, and rice have been reported to have antihypertensive and antioxidant activities due to their content of phytochemical compounds that participate in hormonal regulation mechanisms that help lower blood pressure and other non-transmissible diseases [3,4,5,6].

Peptides derived from food have a high potential regarding the development of nutraceuticals and functional foods due to their specificity and molecular weight [7]. According to Cavazos and Mejia [8], the anti-hypertensive activity of the bioactive peptides presents in cereals with hypotensive effects contribute to preventing cardiovascular diseases. Likewise, it has been discovered that the hydrolyzed proteins and phenolic compounds promote the regulation of oxidative stress and decrease the appearance of associated chronic diseases [9,10]. 

Hypertension has been one of the most important comorbidities that contribute to the development of cardiovascular diseases. Recently, during the pandemic caused by the coronavirus SARS-CoV-2, the most common comorbidities in patients with COVID-19 have been reported, of which hypertension (30%), diabetes (19%), and coronary diseases (8%) stand out [11]. Some recent findings showed an important role of the Renin–angiotensin–aldosterone system (RAAS) in hypertensive patients diagnosed with COVID-19. This is because SARS-CoV-2 utilizes the angiotensin-converting enzyme 2 (ACE2) to unite in the surface of epithelial cells. Thus, controlling the production of ACE2 can mediate the entry of SARS-CoV-2 in the cells [12]. 

Some hypertensive drugs, such as the blocking receptors of angiotensin II (BRA), can modify the expression of ACE2 [13,14], which could decrease the virulence of SARS-CoV-2. The objective of this review is to describe the anti-hypertensive activity present in some bioactive compounds in cereals, wherein activities such as the inhibition of ACE, its participation in oxidative stress, and its consumption could be associated with the prevention of COVID-19. Furthermore, some angiotensin-converting enzyme inhibitors (ACEi) and angiotensin II receptor blockers (ARBs) used in the treatment of hypertensive patients diagnosed with COVID-19 are described.

## 2. Physiopathology of Hypertension

High blood pressure, also known as hypertension, is a public health problem suffered by around 1.3 million adults worldwide. This condition occurs when an elevation in the systolic and diastolic pressure occurs above 140/90 mmHg, respectively [15]. Studies relate the hyperactivation of the renin angiotensin system [16,17], oxidative stress [18], and chronic inflammation [19] as the principal causes in the development of hypertension. Other factors related to hypertension include biochemical processes, such as the increase in the sympathetic activity of the nervous system, the inadequate intake of calcium and potassium, and alterations in the secretion of renin, a hormone related to the elevation in the activity of the angiotensin renin system [20]. In addition, the increased activity of ACE causes a high production of the hormone angiotensin II, as well as deficiencies in vasodilators including vascular inflammatory factors, which promote an alteration in cellular ion channels [20]. 

**Figure 1:**
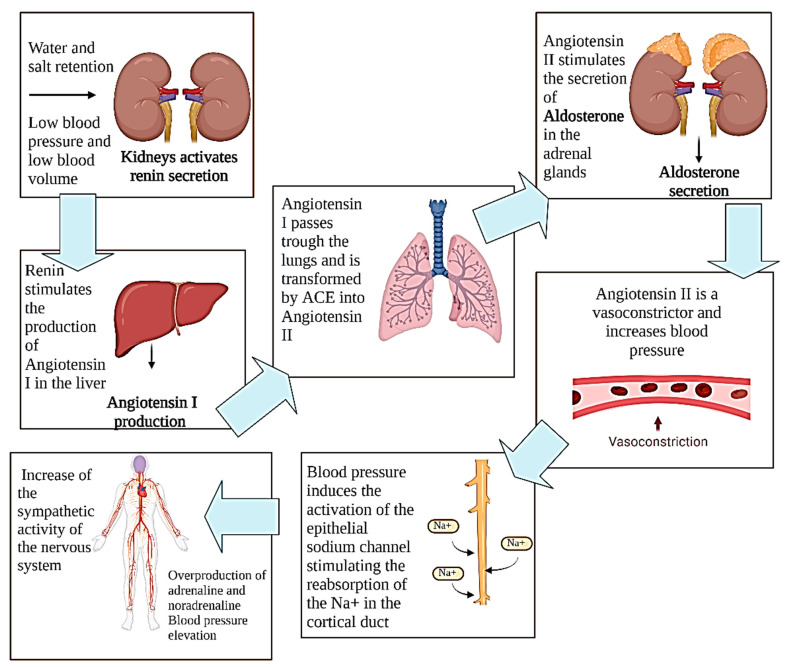
Renin–angiotensin–aldosterone system. Created with BioRender.com (accessed on 5 October 2022).

The RAAS is the principal mechanism that affects the regulation of blood pressure. An increase in the renin hormone caused by an increment in the intake of sodium provokes the stimulation of the production of the physiologically inactive hormone called angiotensin-I (Ang-I), which is converted into angiotensin II (Ang-II) due to the angiotensin-converter enzyme (ACE-I). Ang II is a vasoconstrictor that stimulates the production of aldosterone, which causes an increase in blood pressure through the retention of sodium and water. This induces the activation of the epithelial sodium channel stimulating the reabsorption of the Na+ in the cortical duct (Figure 1) [17,21].

Although the potential of antihypertensive drugs to lower blood pressure in individuals with hypertension has been shown, lifestyle habits, such as regular exercise and healthy eating, have also been reported to have a positive effect on blood pressure control [22]. Some mechanisms used by the bioactive compounds present in food, mainly polyphenolic compounds to reduce hypertension, include the reduction in the levels of the vasoconstrictor molecule I and the increase in the antioxidant glutathione [23], which improve the production of vasodilator factors such as oxide nitric [24] and inhibit the expression of proangiogenic factors such as vascular endothelial growth factor [25]. 

Therefore, a better understanding of the hormonal mechanisms that control high blood pressure could clarify the causes and effects that a drug treatment combined with a diet rich in cereals could have on the control of hypertension, effectively reducing inflammation and oxidative stress and strengthening the immune system during the COVID-19 crisis.

## 3. Hypertension: Main Comorbidity in Patients with COVID-19

COVID-19, caused by the SARS-CoV-2 virus, is an infectious disease that has provoked a sanitary crisis worldwide. The pathogenesis of the SARS-CoV-2 virus starts by means of the union of the protein of the viral peak with the target receptor of ACE2, which facilitates the internalization of the virus within the host cells. It was reported that SARS-Cov-2 is a virus whose tropism is based on the use of ACE2 to unite the epithelial cells of the organism [26,27]. The ACE balances blood pressure and converts angiotensin I into angiotensin II with a vasoconstrictive function and at the same time facilitates the degradation of a vasodilator termed bradykinin [28]. The control over these hormonal processes balances the health of hypertensive patients. However, a combination of other diseases makes it difficult to control and, in many cases, can worsen the evolution of each illness. Therefore, the initial reports suggest that hypertension, diabetes, and cardiovascular diseases are the most frequent comorbidities in COVID-19 [29]. 

The ACE2 can change the balance of the RAAS by means of the conversion of Ang II to Ang (1-7). Therefore, hypertension and COVID-19 have developed into a recent concern over the susceptibility of patients with hypertension to develop COVID-19, as it increases the severity of the illness and the use of ACEi and ARBs [30]. 

The inhibitors utilized in the treatment of hypertension increase the expression of ACE2 on the cellular surface and can increase the expression of the intestinal messenger ribonucleic acid (ARNm) of ACE2. Although there are few data concerning the effects of these drugs regarding the expression of the ARNm of ACE2 in the pulmonary epithelial cells, there exists the concern that the patients who take these treatments can encourage the contraction of the virus [31].

An optimum immune response is the key to maintaining control over infectious and non-infectious diseases. An increase in the intake of whole cereals rich in fiber and polysaccharides is associated with a reduction in PCR-hs (a marker used to predict cardiovascular events in patients with atherosclerosis via Polymerase Chain Reaction) [32]; decreased interleukin-6 (IL-6) [33], which is produced in response to infections and tissue damage; and tumor necrosis factor alpha (TNF-α), an inflammatory cytokine produced by macrophages/monocytes during acute inflammation [2]; therefore, cereals reduce the risk of suffering illnesses predicted by inflammation such as cardiovascular diseases [34] and diabetes type II [35].

Since blood pressure is difficult to control, the most widely used resources involve identifying drug targets to effectively control and manage blood pressure in hypertensive patients.

## 4. Anti-Hypertensive Drugs and Their Use in the Treatment of COVID-19

The use of ACEi and ARBs have been associated with a decrease in the mortality of a hospital population diagnosed with COVID-19 and with a reduction in the hospital in-patient stay observed with a greater effect in patients with hypertension [36]. 

However, it has been shown that ACEi and ARBs could facilitate the entry of the virus into the host cell and increase the chances of infection or its severity, although there are no conclusive studies [37]. In a study of 187 patients with COVID-19 (the mean age was 58.5 years), it was observed that the mortality of those treated with ACEi/ARBs did not show a significant difference with those who were not treated with ACEi/ARBs [38]. 

Martínez-del Río et al. [39] reported that the use of ACEi and antagonists of the angiotensin receptor 2 (ARA2) in elderly patients does not increase the risk of death or the use of assisted ventilation, but the use of these drugs overexpress ACE2 and increases the risk of infection. This enzyme acts by inhibiting angiotensin 2 and increasing the production of angiotensin 1–7 with anti-inflammatory and vasodilator effects [40], which have been found in greater levels in persons that have survived respiratory stress than in persons who have perished [41].

Braude et al. [36] reported the influence of ACEi and ARBs on mortality in 1371 patients with a mean age of 74 years diagnosed with COVID-19. The results showed a significant reduction in hospital stay. This was because ACEi decreases the production of ACE2, as it blocks the conversion of ACE1 to ACE2, and the ARBs block the receptor of angiotensin II type I impeding the actions of ACE2 concerning pulmonary vasoconstriction and endothelial permeability, thereby diminishing the injury at the pulmonary level. Therefore, the use of ACEi could decrease the progression and mortality of patients with COVID-19.

One strategy to treat infection with COVID-19 is to inhibit the entry of SARS-CoV-2 in the host cell through the receptors of ACE2 [42]. Consequently, the positive regulation of ACE2 in infected patients with SARS-CoV-2 could be clinically useful due to the vascular protection provided by the activity of angiotensin 1–7, thereby diminishing the effects of angiotensin II on vasoconstriction and the retention of sodium [43].

**Figure 2:**
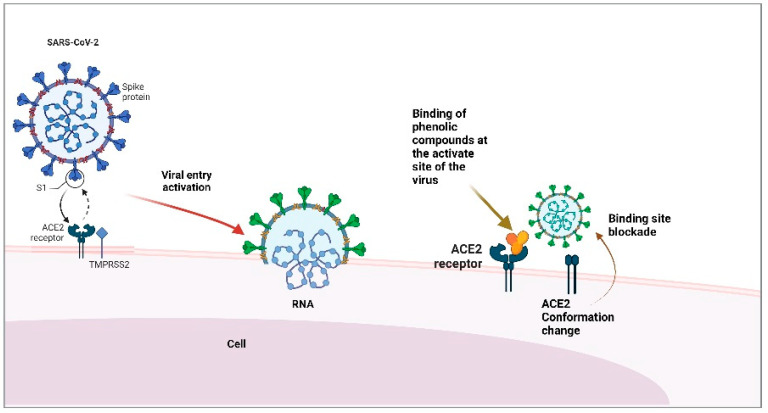
Phenol binding to the ACE2 receptor and protein S blockade of SARS-CoV-2. Created with BioRender.com (accessed on 28 August 2022).

Bioactive compounds are valuable for drug development and adjunctive therapies for the related infection. These compounds can act as preventive agents or as treatment accelerators. Flavanones, flavones, and saponins are some natural ACE2 inhibitors [44,45]. Saponins can inhibit the binding of COVID-19 protein S to ACE2 receptors [46] (Figure 2).

**Figure 3:**
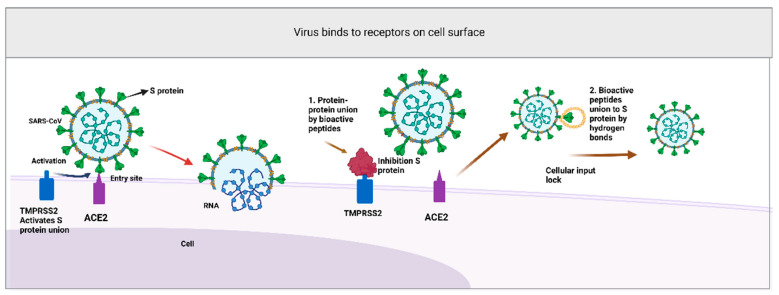
Peptide activity on SARS-CoV-2: (1) Inhibition of TMRPSS2 by bioactive peptides blocks priming of virus S proteins. (2) Inhibition of protein S by amino acid residues through hydrogen bonds prevents SARS-CoV-2 virus’ entry. Created with BioRender.com (accessed on 28 August 2022).

The peptide inhibitors that are used in the treatment of diverse diseases could also be potential agents against COVID-19. The bioactive peptides with unique sequences of amino acids can mitigate the inhibition of the transmembrane proteases and serine type II (TMPRSS2), a gene regulated by androgens, for the priming of the viral protein peak, furin split, and the members of the renin–angiotensin–aldosterone system (RAAS). On the other hand, it has been shown that the inhibition of virus replication could be mediated by hydrogen bonding through the binding of amino acid residues [47,48] (Figure 3). 

The peptides of a food origin can perform diverse bioactivities, including antiviral activities, depending on their characteristics and sequencing [49]. Therefore, the peptides derived from cereals could serve as inhibitors of multiple processes regarding the entry into the host cell and the viral replication of SARS-CoV-2. Diverse epidemiological studies highlight the importance of the consumption of diets rich in cereals and products of a natural origin that help to protect against hypertension and viral diseases such as COVID-19 [33,50,51]. 

## 5. Cereals as a Source of Compounds with Anti-Hypertensive Activity

The flavonoids and phenolic acids present in cereals have an ACE-inhibitory capacity mainly associated with blood pressure-lowering effects due to their antioxidant capacity [52]. The regulation of reactive oxygen species, the reduction in oxidative stress, and the formation of zinc chelates are factors that promote the lowering of blood pressure [53,54]. In Table 1, the in vivo or in vitro antihypertensive mechanisms of phenolic compounds present in some cereals are described. 

In addition to phenolic compounds, studies have been conducted on multiple candidates for antihypertensive peptides, which, because of their biological activity, can be generated or incorporated into functional foods. Table 2 summarizes studies highlighting cereal peptides and proteins with antihypertensive activity. Proteins with a molecular weight lower than 1 kDa favor their entry through cell membranes enabling their absorption and circulation [64]. Hydrolyzed proteins with high levels of proline and other amino acids contribute to enzyme inhibition by chelation with zinc at the active center of the enzyme and its interaction with hydrophobic sites. Therefore, the ionic interaction between amino acids and zinc enhances the competitive activity for the catalytic sites of ACE [65,66]. Since there are antihypertensive peptides from cereals rich in proline and other hydrophilic amino acids related to the S protein of SARS-CoV-2, they could serve as multi-target inhibitors against host cell entry. The antihypertensive rice bran tripeptide Tyr-Ser-Lys, reported by Wang et al. [67], has two aliphatic amino acids in its chain with a hydroxyl in the C-terminal chain; thus, it could have antiviral effects. Similarly, the peptide Gly-Phe-Pro-Thr-Leu-Lys-Ile-Phe—reported by Gangopadhyay et al. [68]—in barley flour presents four hydrophilic amino acids, increasing the chances that it will be coupled to the S protein of the virus that causes COVID-19. An in silico study showed that some oligopeptides from barley, oats, wheat, and soybeans (PISCR, VQVVN, PQQQF, and EQQQR) were identified as potential binders of the SARS-CoV-2 spike protein receptor-binding domain (RBD) [69]. This feature is also observed in short-chain peptides isolated from cereals [70]. Antihypertensive peptides generally contain amino acid residues at the C-terminus or N-terminus. The presence of tyrosine, phenylalanine, tryptophan, proline, lysine, isoleucine, valine, leucine, and arginine present in the peptides influence the binding of the ACE substrate or inhibitor [71]. According to the reported studies, an association has been established between the presence of bioactive compounds and the ACE-inhibitory mechanism and this could have a significant impact on the active sites of SARS-CoV-2.

### 5.1. Rice

Wild rice (*Zizania* spp.) is one of the cereals that has presented anti-hypertensive, antiallergic, and immunomodulating activities, which are associated with the phenolic acids, flavonoids, and other phytochemicals with antioxidant properties that aid in the prevention of chronic illnesses [89,90]. Okarter and Liu [91] report that the low incidence of chronic diseases in regions where rice is consumed is related to the presence of phytochemical antioxidants in this cereal. Consequentially, these studies suggest the potential use of rice and its by-products in the prevention or contributory treatment of non-transmissible diseases such as hypertension.

Gong et al. [89] quantified the total phenolic content and flavonoids in different varieties of rice, such as black rice, red rice, whole rice, and plain rice. They reported concentrations of 1159, 669, 108.7, and 58.88 mg of Gallic Acid Equivalents (GAE)/100 g. With respect to the total content of flavonoids, the authors reported 1503, 598.2, 77.94, and 26.52 mg Quercetin Equivalents (QE)/100 g in black rice, red rice, whole rice, and plain rice. Deng et al. [92] demonstrated the antihypertensive effects of wild rice (*Zizania latifolia)* in spontaneously hypertensive rats, attributing these effects to the influence of the polyphenol content, principally quercetin, due to previous studies that have demonstrated that this compound reduces blood pressure and, moreover, since it presents protective effects against cardiovascular diseases. Table 1 shows the main mechanism used in the ACE inhibition of some phenolic compounds derived from cereals such as rice. The phytochemical composition of wild rice is so complex that the decrease in hypertension could be related to the synergic effects of bioactive compounds such as polyphenols and bioactive tripeptides [92,93]. 

Michelke et al. [72] evaluated possible ACE inhibitor peptides found in hydrolyzed whey, soy, and rice protein. The evaluation of ACE inhibition was performed in different ACE systems such as human plasma, venous endothelial cells from human umbilical cord, rabbit lungs, and rat aortic rings. The IC50 values observed in the soybean and rice peptide mixtures were approximately 2 to 2.5 times higher than the IC50 value of the serum-derived peptides. Therefore, the best ACE-inhibitory activity was from the serum peptides consisting of isoleucine and tryptophan.

Some studies have shown the effectiveness of dipeptides made up of isoleucine and tryptophan (IW) in decreasing ACE, showing anti-inflammatory and antioxidant activities in endothelial cells [94,95]. Lunow et al. [96] mention that the IW dipeptide acts as a competitive and selective inhibitor for the C-Terminal of ACE in plasma. 

Jan-on et al. [54] demonstrated that virgin rice bran oil prevents hypertension induced by the L-NG-nitroarginine methyl ester (L-NAME) in rats, improving the hemodynamic alterations, as well as the reduction in oxidative stress and vascular inflammation. This suggests that these activities could be mediated by the content of unsaturated fat, antioxidants, phytochemicals such as g-oryzanol, phytosterols, and tocopherols, which possess antioxidant activities and provide vascular and inflammatory protection.

On the other hand, rice bran presents a high concentration of biologically active compounds that are important for human health, of which are found cellulose, hemicellulose, pectin, arabinoxylan, lignin, β-glucan, polyphenols, γ-oryzanol, β-sitosterol, vitamin B9, vitamin E, tocopherols, micronutrients (such as calcium and magnesium), and essential amino acids (such as arginine, cysteine, histidine, and tryptophan) [97].

Due to the high content of nutrients, a diet rich in rice increases immunological, antioxidant, anticancer, and antidiabetic activities, protecting the organism against multiple diseases [98]. Therefore, the use of these compounds and their different functions as collectors of free radicals, antiallergy agents, antiatherosclerosis agents, anti-influenza agents, anti-obesity agents, and antitumor agents offer protection against numerous chronic diseases and degenerative diseases in humans, including hypertension and some cases that could interfere with the infection of COVID-19.

### 5.2. Barley

In barley (*Hordeum vulgare* L), phytochemical concentrations have been reported in relation to a reduction in heart disease, colon cancer, gallstones, and cardiovascular illnesses [99]. The Food and Drug Administration reported that the intake of barley is related to a decrease in cardiovascular diseases [100], such as chronic coronary diseases, due to the decrease in plasma cholesterol promoted by β-glucans from hulled barley, which promote the excretion of fecal lipids [101]. 

Some of the properties that are attributed to barley for reducing the risk of cardiovascular diseases such as hypertension are related to their different bioactive components, which include peptides and ACE-inhibitory proteins [102]. However, some studies mention that the high inhibition of ACE is principally stimulated by the combination of components that come from antioxidants [103], peptides [68], or phenolic compounds [104].

Different authors have also demonstrated the great variety of bioactive compounds originating from barley [57,68], among which the most utilized are inhibitors of ACE. The total concentration of phenolic acids ranges between 604 and 1346 mg/g [105]. Kim et al. [106] studied the content of 127 varieties of barley with and without husks and they found that the flavonoid content ranged from 62–300.8 mg/g. Andersson et al. [107] studied 10 varieties of barley and found that the concentration of phytosterols ranges between 820 and 1153 mg/g. With respect to anthocyanins, the most common found in barley is the cyanidin 3-glucosidic type (214.8 mg/g) [108]. On the other hand, the lignans are the most studied polyphenols in barley, whose concentration ranges between 6.6 and 541 mg/100 g [109].

The peptides obtained from barley also present inhibitory effects towards ACE. The effects of the peptides occur principally because of the presence of hydrophobic peptides in the C-terminal chain of the peptide that are united in the active sites of ACE [68]. The presence of anthocyanins and polyphenols extracted from whole grains and seedlings of barley, respectively, have also been studied as potential inhibitors of ACE, presenting competitive and non-competitive inhibitory mechanisms. Some polyphenols show a non-competitive inhibition of ACE when a structural difference with the natural substrate of ACE is produced (Table 1) [57,58].

In addition to phenolic compounds and inhibitory peptides, the soluble fiber in barley and other cereals has an important role in human health. A study by Behall et al. [110] observed a reduction in systolic and diastolic blood pressure in middle-aged men and women after a 5-week integral diet. Fiber has anti-inflammatory effects, and in adults with asthma, an average fiber intake of 5 g/day plus a controlled mineral-rich diet is inversely associated with the eosinophilic inflammation of the respiratory tract and pulmonary function [111,112]. Epidemiological studies in humans have demonstrated that fiber can promote health and prevent chronic diseases, especially those related with inflammation [113], which could improve the cognitive function of people infected with COVID-19 [113]. Therefore, the intake of dietary fiber can support antiviral and immunosuppressive therapeutic treatments, thereby ameliorating the suffering of COVID-19 [114].

### 5.3. Corn

Across the globe, there are different varieties of corn, which is rich in fiber, vitamins, minerals, phenolic acids, flavonoids, sterols, and a great variety of phytochemicals [115]. There are reports that indicate that corn is one of the cereals with the highest availability of nutrients, mainly β-carotene and α-tocopherol, which suggests that it may be the most suitable for biofortification [116]. However, this may depend on the pigments in the grain. Blue, red, and purple corn have a higher concentration of anthocyanidins; in Chinese purple corn, approximate concentrations of 256.5 mg of cyanidin 3-glucoside/100 g at a dry weight have been reported, while in American corn, the anthokinin content ranges from 54 to 115 mg/100 g per sample [117,118]. Yellow corn is rich in carotenoids with a concentration of 0.823 mg/100 g per dry weight of corn [119]. Violeta et al. [116] have reported concentrations of 26.9 μg/g of β-carotene and 27.2 μg/g of α-tocopherol in dark orange corn grains, while in dark red corn, they were 2.51 and 4.95 μg/g, respectively, and in red corn, they only reported a concentration of α-tocopherol of 4.87 μg/g. Pigmented genotypes have shown a strong antioxidant capacity using DPPH and TEAC techniques [120]. In black corn, a higher antioxidant activity has been reported than in yellow and white corn [121]. According to reports, the type of phenolic compound and/or flavonoids are associated with grain pigmentation. The bioactive compounds have been related to antioxidant [122], anticancer [123], antimicrobial, and anti-viral activities [124]. The anthocyanin content differs by the variety of corn; in pink corn, it is approximately 12.74 mg of cyanidin 3-glucoside/100 g at a dry weight, while in black corn, the anthocyanin content is 304.5 mg of cyanidin 3-glucoside/100 g at a dry weight [118]. Corn has the highest antioxidant activity with 181.4 μmol equivalents of vitamin C/g per grain compared to cereals such as rice that have 55.77 μmol equivalents of vitamin C/g per grain, wheat with 76.70 μmol equivalents of vitamin C/g per grain, and oats with 74.67 μmol equivalents of vitamin C/g per grain [125].

Mellen et al. [126] carried out a metanalysis regarding the intake of whole grains and clinical cardiovascular events. According to their estimates, the consumption of whole cereals reduces the risk of suffering cardiovascular diseases by 21%. Similarly, this has been related to a decrease in the risks of suffering chronic diseases such as diabetes type 2 [127], obesity, some cancers [128,129], and cardiovascular diseases [130]. Wu et al. [74] evaluated the antihypertensive activity of ACE inhibitor peptides from corn germ using the hydroenzymatic lysis method with alkaline protease that allows for the production of a high concentration of inhibitor peptides. They carried out an ultrafiltration that allowed them to obtain smaller peptides of 6 kDa, increasing the IC50 of the inhibitory activity of ACE and demonstrating that the smaller the size the better absorption, according to the authors [7,75]. 

It has been reported that the peptides extracted from corn germ flour promote the balance between vasoconstrictor factors, vascular endurance, and the reduction in the level of renin and Angiotensin II, thus controlling blood pressure [73,74]. Huang et al. [75] demonstrated the antihypertensive effect of the peptides of corn in spontaneously hypertensive rats. They reported that two types of mechanisms of action exist in the peptide inhibitors of ACE: those that compete with the availability of the substrate of ACE and those that combine the bioactivity of ACE to inhibit its enzymatic activity. These are normally made up of more than four amino acids and from two to three amino acids. It is shown in this study that the molecular size of the inhibitory peptide of ACE plays an important role in its inhibitory activity because the peptides less than 3 kDa had an inhibition four times greater than the peptides of 5 kDa. In a dipeptide (Ala-Tyr) isolated from a hydrolyzed corn gluten flour, an IC50 of 82.92% was observed; therefore, due to its size, it is a potential ACE inhibitor [131]. Some peptides and proteins derived from cereals with antihypertensive activity are shown in the Table 2.

Duru [132] showed that the minerals and phytochemical content present in corn husks contribute to multiple health benefits. Among the most abundant minerals that can be found are calcium, sulfur, and potassium, which contribute to nerve and muscle regulation. This is the case for calcium; sulfur is present in different amino acids and potassium plays a part in the acid–base balance and osmotic regulation. As a consequence, a modification of the diet that includes the consumption of corn could be a strategy to prevent cardiovascular diseases and infectious diseases such as COVID-19. 

### 5.4. Wheat

Wheat (*Triticum* spp.) has been used for the elaboration of basic foods since time immemorial and is highly essential in human nutrition, providing 55% of starch and more than 20% of food calories. Clinical studies have demonstrated that the regular consumption of wheat is associated with a reduction in chronic diseases, specifically the intake of dietetic fiber and other bioactive compounds [4].

Wheat is a rich source of diverse phytochemicals, among which are phenolic acids, terpenoids, tocopherols, and sterols [133]. The concentration of phenolic acids in whole wheat ranges between 200 to 1200 mg/g in dry weight [134]. The type of milling and the use given to this cereal has a great impact on the composition of the bioactive compounds and, thus, the health benefits as well as the improvement of the functions of the colon, those against cancer, those that protect against obesity, those that promote weight loss, and those that mitigate cardiovascular diseases [4,135].

Zhang et al. [66] isolated peptides from wheat gluten for their potential use as ACE inhibitors, showing the importance of generating gluten hydrolysates to increase their benefits, especially for the celiac population.

Besides gluten, wheat germ has widely been studied because of its high protein content. Diverse studies have demonstrated that the peptides isolated from wheat germ and some isolated from wheat gluten, such as VPL (Val-Pro-Leu), WL (Trp-Leu), WP (Trp-Pro), and IAP (Ile-Ala-Pro), present antihypertensive effects principally for ACE inhibition, which is caused principally by the high presence of hydrophobic amino acids such as proline and tryptophan (Table 2) [8,78,79]. 

Asoodeh et al. [77] performed a characterization of ACE-inhibitory peptides from wheat gluten protein hydrolysates through the use of trypsin. The sequences with the highest inhibitory activity were Ile-Pro-Ala-Leu-Leu-Lys-Arg and Ala-Gln-Gln-Leu-Ala-Ala-Gln-Leu-Pro-Arg-Met-Cys-Arg; as in most inhibitory peptides, this activity is influenced by the peptides’ structure, since some peptides that have tryptophan, tyrosine, phenylalanine, and proline residues and hydrophobic amino acids in the C-terminal sequence show greater inhibitory activity towards ACE [136].

Besides the extraction and evaluation of inhibitory peptides, Gammoh et al. [80] demonstrated that the isolation of phenols from protein fractions in wheat flour increased antihypertensive activity in an in an vitro model, alongside increasing antioxidant properties and decreasing allergenicity.

Recently, studies have demonstrated the capacity of polysaccharides to increase the immune response to infectious diseases. In cells such as macrophages, the polysaccharides activate the protein tracts, stimulating the control processes of the immune response [137]. Therefore, the polysaccharides of wheat induce the expression of cytokines, activating macrophages and increasing the phagocytotic activity [138,139]. Thus, the polysaccharides activate the important immunosuppression tracts for the treatment of persons infected with COVID-19 because they stimulate the production of anti-inflammatory substances, which could apply to the treatment of grave cases [137].

### 5.5. Oats

Oats (*Avena sativa*) are a whole cereal that provide proteins, unsaturated fatty acids, vitamins, minerals, dietetic fiber, and phenols such as the avenanthramides [140]. Soycan et al. [141] determined the concentration of phenolic acids and avenanthramides in commercial products of oats and showed that there was a greater concentration of these compounds (1518.6 μg/g) compared to oat bran (626.3 μg/g). Different bioactive compounds have been reported in oats, such as phenolic compounds, with a concentration between 180 and 576 mg Routine Equivalents (RE)/100 g. As to the phytosterols, oats present a concentration between approximately 35 and 68.2 mg/100 g. On the other hand, the tocopherol content (vitamin E) ranges between 0.5 and 3.61 mg/100 g [142].

Diverse studies mention that a regular consumption of oats reduces cholesterol [5,143], improves the sensitivity of insulin [144], and controls blood pressure [145]. Soyca et al. [141] reported a concentration of total phenolic acids of 39.5-62.75mg/100 g per sample. In this study, it is mentioned that ferulic acid is the principal component present in commercial oats, consisting of 58-78.1% of the total compounds. Ferulic acid presents antioxidant activities that can prevent chronic diseases [146]. It has been demonstrated that avenanthramides offer health benefits such as antioxidative properties that can help protect against cardiovascular diseases [147]. 

Few studies have investigated the benefits offered by oats in hypertension. However, their positive effects on cardiovascular diseases have not been discarded. Wang et al. [81] evaluated the ultrasonic pre-treatment of the protein in oats and its activity as a protein inhibitor of ACE, utilizing the enzymatic pre-treatment with ultrasound for the improvement of the hydrolysis of proteins and the process of enzymolysis for the liberation of peptides less than 3kDa. The results showed that the ultrasonic energy, the duration of treatment, and the time of enzymolysis greatly influenced the hydrolysis grade and inhibitory activities of the ACE of the peptides. They showed that the inhibition of ACE provoked by the peptides had an increase of 32.1 to 53.8% compared to samples without ultrasonic treatment. According to the authors, the rate of enzymatic hydrolysis after ultrasonic pre-treatment was due to the increase in the affinity between the alcalase and the isolated protein. Alcalase is a specific endonuclease enzyme that combines exposed hydrophobic sides, which could have brought about an increase in the production of inhibitor peptides of ACE, provoked by the high grade of the hydrolysis it promoted [148]. 

Besides the protein inhibitors of ACE, the soluble fibers such as the β-glucans of oats have been widely studied, demonstrating prebiotic effects and improving glycemic control and regulating blood pressure [149,150]. Maki et al. [151] evaluated the effect of the consumption of foods that contain the β-glucan from oats in blood pressure. The study consisted of a controlled randomized clinical trial, which was double blinded, where 97 men and women, with a mean age of 63 years, systolic blood pressure of 130–179 mmHg, and/or a diastolic blood pressure of 85–109 mm Hg were assigned to consume foods containing oat β-glucan or control foods for 12 weeks. Although the results did not show a significant difference in terms of the decrease in blood pressure between the groups, the decrease in blood pressure significantly decreased both the systolic (8.3 mm Hg, *p* = 0.008) and diastolic (3, 9 mm Hg, *p* = 0.018) pressure in the subjects with a body mass index above the mean (31.5 kg/m^2^) compared to the control groups.

The extracts of β-glucans produce immunomodulatory effects and pulmonary cryoprotections, which could have therapeutic implications in patients with COVID-19. In the same way, these could reduce oxidative stress and activate macrophages [33]. 

McCarty and DiNicolantonio [152] recently described the potential role of β-glucan as a natural nutraceutical to boost the response of interferon type 1 to RNA viruses such as the influenza and the coronavirus. Therefore, the intake of oat products provides a rich source of phytochemicals that provides health benefits such as decreasing high blood pressure and influencing the immunotherapies against infections such as COVID-19 due to the presence of inhibitory peptides of ACE and of β-glucans.

### 5.6. Millet

Millet includes numerous species that are not related genetically. However, it contains various phytochemicals, phenolic compounds, phytosterols, policosanols, and bioactive peptides [153]. Chandrasekara and Shahidi [154] evaluated different varieties of this cereal that presented approximate concentrations of hydroxybenzoic and hydroxycinnamic acids and their by-products from 9.3 to 62.2 µg/g and 9.1 to 173 µg/g of defatted flour, respectively, both in their free forms. As to flavonoids, this cereal contains from 2 to 100 mg/g, which differs because of the variety of the species [153] 

The protein of foxtail millet (*Setaria italica Beauv*) can have physicochemical and physiological properties. Some studies have found that foxtail millet presents antioxidant activities, reduces the levels of cholesterol, and can present anticancer effects [155,156].

Furthermore, foxtail millet presents antihypertensive effects. Studies reported the inhibitory capacity of the ACE of hydrolyzed proteins derived from this cereal [87]. The consumption of whole grains can reduce blood pressure. Hou et al. [88] reported that the consumption of 50 g of whole grains of pulverized foxtail millet extruded in the form of bread or millet pancakes for 12 weeks showed a significant reduction in SBP of 133.61 and 129.48 mmHg, as well as a reduction in the mass index and body fat in 45 middle-aged hypertensive patients. However, Chen et al. [87] showed the best results with respect to decreasing blood pressure. In this study, they used spontaneously hypertensive rats. They showed that a diet of 200 mg of peptides per kg of body weight for four weeks reduces blood pressure via the intake of raw samples and in extruded and fermented samples with *Rhizopus oryzae*. Compared to the extruded and fermented samples, the raw samples caused a greater decrease in blood pressure with a reduction of 28.3 mmHg in PAS. As to the extruded and fermented hydrolyzed proteins, there was a reduction of 24.8 and 13.6 mmHg, respectively. A controlled group treated with captopril had a reduction of 23.6 mmHg.

Therefore, the consumption of foxtail millet protein, specifically hydrolyzed, raw, and extruded millet protein, improves hypertension due to the antioxidant and anti-inflammatory properties whereby vascular conditions can be regulated gradually (Table 2) [157]. In both studies, the levels of ACE and Ang II decreased, which could indicate that the antihypertensive mechanism of foxtail millet consists of inhibiting the activity of the ACE in the serum of subjects with slight hypertension. The antihypertensive effects produced by cereals are related to the improvement in the endothelial function that is achieved by inhibiting the effects of vasoconstrictors such as Ang II, inducing vasodilatation through nitric oxide, and affecting the vasorelaxation tracts involved. Along with the previously mentioned cereal, the consumption of millet can aid the modulation of immune functions, which helps to protect against the COVID-19 ailment [158]. 

### 5.7. Rye

Among cereals, rye (*Secale cereale* L.) contains the highest concentration of dietetic fiber, which is composed of arabinoxylan, cellulose, β-glucan, fructans, and lignin. Arabinoxylan is the most abundant fiber in rye (7.6–12.1% of the dry grain weight) [159]. Pihlava et al. [160] reported 0.5, 4.6, and 20.5 mg/100 g of dry weight of total flavonoids present in the fine flour of rye, whole rye flour, and rye bran, respectively. As to the quantity of anthocyanins, the authors reported 0.15 mg/100 g in rye bran, 0.18 mg/100 g in whole rye flour, and 0.026 mg/100 g in fine rye flour. They also reported 66.3, 15.5, and 291.6 mg/100 g in the dry weight of alkylresorcinols in whole rye flour, fine rye flour, and rye bran, respectively. 

There is important evidence within the studies of the physiological effects of rye foods with possible health benefits, such as the positive effects on tumors in prostate cancer [2], antihyperglycemic properties, and antihypertensive activities [86]. Zhao et al. [86] evaluated the concentration of inhibitors of the ACE of different bakery products starting with rye sourdough. They reported eight ACE-inhibitory tripeptides. The dominant tripeptide was IPP (Ile-Pro-Pro) with 58 to 73 mmol/kg. Moreover, the peptide that showed the greatest inhibition of ACE was LPP (Leu-Pro-Pro) (57 mmol/L), which is characterized by the presence of leucine, an amino acid with a greater hydrophobicity, which is a principal characteristic of the inhibitors of ACE.

Rye grains are a source of diverse phytochemicals such as phenolic acids, lignans, and alkylresorcinols [160]. Multiple studies have demonstrated the capacity of the secondary metabolites of plants to generate antiviral activities besides the importance of phytochemicals against SARS-CoV [161,162]. There are studies that link the effectiveness of dietary fiber to the prevention of diseases related to lifestyle such as hypertension [163,164]. Dietary fibers reach the colon and produce short-chain fatty acids, which are released into the circulation to reach the organs involved in the regulation of hypertension [165]. Due to the high content of dietary fiber, proteins, and various bioactive compounds, rye can enhance immunomodulatory and antihypertensive activities.

### 5.8. Sorghum

Sorghum (*Sorghum* spp.) contains tannins, phenolic acids, anthocyanins, and phytosterols. These phytochemicals have the potential to provide a significant impact on human health, promoting cardiovascular health by reducing the plasma levels of lipoproteins of a low density and hepatic cholesterol [166]. Sorghum contains benzoic acids and cinnamic acids, which range from 16 to 131 mg/g and from 41 to 444 mg/g, respectively [167]. 

Anthocyanins are the most studied flavonoids in sorghum; Awika et al. [168] reported that the anthocyanin content in black sorghum bran is three to four times higher than in whole grain and had at least twice the anthocyanin levels (10.1 mg/g) in comparison with red sorghum (3.6 mg/g). The quantitative data of the phytosterols present in sorghum are limited, although approximate contents of 44 to 72 mg/100 g have been reported [169,170]. 

The generation of ACE-inhibitory peptides has been carried out in different forms. Most of these techniques were based on the production of peptides from food proteins via enzymatic hydrolysis [66]. Wu et al. [84] developed a kinetic method that describes the enzymatic hydrolysis of the protein of sweet sorghum grain utilizing alcalase to purify ACE-inhibitory peptides (Table 2). The authors demonstrated that 19% hydrolysis exhibited the strongest inhibitory activity of ACE. On the other hand, they obtained a tripeptide inhibitor composed of Threonina (Thr)-Leucine (Leu)-Serine (Ser), which, due to the serine union at the C-terminal of the chain, manages to interact in the peak protein subunits (S1 and S2) of ACE, thereby achieving its inhibition. Some studies explained the relationship between the structure and the activity of the inhibitory peptides of ACE, which are influenced by the C-terminal and the presence of hydrophobic amino acids or aromatic residues such as Tryptophan (Trp), Tyrosine (Tyr), Proline (Pro), and Phenylalanine (Phe). However, this structure-activity relationship has not been completely established [148].

The polyphenols have an ample antiviral activity against diverse groups of viruses such as influenza A (H1N1), hepatitis B and C (VHB/VHC), herpes simplex 1 (VHS-1), human immunodeficiency virus (HIV) and, recently, the virus that caused the COVID-19 disease (SARS-CoV-2) [171].

Besides their antiviral capacity, the phenolic compounds can also present antihypertensive activity. Irondi et al. [61] analyzed raw and toasted red sorghum grain flour (150 and 180 °C) to determine the inhibitory activities of different enzymes including ACE. They found that the raw grains showed high inhibitory activities (19.64 μg/mL) because of the high presence of phenolic acids (gallic, chlorogenic, caffeic, ellagic, and p-coumaric) and flavonoids (quercetin, luteolin, and apigenin), as increasing the temperature when toasting decreases the presence of phenolic compounds and, consequentially, causes a decrease in inhibitory activity, with an IC50 in the grains roasted at 150 °C of 20.99 μg/mL and in the grains roasted at 180 °C of 22.81 μg/mL. Therefore, the parallel decrease in the inhibitory activity of the enzymes and the phenolic composition of the grains with the increase in the toasting temperature suggests that the phenolic acids and the flavonoids could be the principal inhibitors of the enzymes of the grain. 

In this way, sorghum is a cereal with high potential to control hypertension and, in some cases, its consumption could reduce the probability of viral infection by SARS-CoV-2 due to its high phytochemical content. In general, this cereal seems to have a great potential to form part of a healthy diet and its consumption as grains or as food products could reinforce the bioavailability of nutrients to prevent chronic diseases and infections.

## 6. Conclusions

Different components of cereals have been characterized, such as anthocyanins, flavonoids, phenolic acids, proteins, and fibers, which have biological activities that help prevent or control hypertension acting on the RAAS, inflammation, and oxidative stress. According to the studies reported in this review, pigmented raw rice exhibits the greatest ACE inhibition. In an in vitro study, raw rice was shown to inhibit up to 97% of ACE. This activity is related to the reduction in oxidative stress and the reduction in NOS, caused by the presence of phenolic compounds such as proanthocyanidins. In silico studies showed that peptides derived from oats, made up mainly of aromatic amino acids, can inhibit up to 96.5% of ACE. The presence of this type of amino acid is also related to the ability to inhibit the TMPRSS protease of the host to prevent the entry of the SARS-CoV-2 virus. ACE inhibitor drugs (ACEi) and angiotensin II receptor blockers (ARBs) participate in processes that regulate the expression of ACE2, thus being useful in the treatment of patients who developed SARS-CoV-2. Ultimately, this review highlighted the mechanisms used by bioactive compounds in cereals to lower blood pressure and how these processes could be involved in reducing the degree of COVID-19 infection.

## Figures and Tables

**Table 1:** Phenolic compounds derived from cereals with antihypertensive activity.

Food	Main Phenolic Compound	Test	IC_50_ or % IECA	Decrease BP	Main Mechanism	Reference
Virgin rice bran oil	Sterols, tocopherols, and tocotrienols	in vivo	ND	25.5%	Regulation of NOS and reduction in oxidative stress	[54]
Raw rice	Phenol acids Flavonoids	in vitro	97%	ND	Competitive inhibition of ECA	[55]
Rice bran hydrolysate	Phenolic compounds	in vivo	ND	31.5%	Endothelium-derived hyperpolarizing factor-mediated vasorelaxation and L-type Ca 2+ channel-mediated vasoconstriction	[56]
Barley seedlings	Polyphenols	in vitro	66.5%	ND	Non-competitive inhibitors of ECA and formation of chelates with ions of zinc	[57]
Barley whole grain	Anthocyanins	in vitro	8770 µg/mL	ND	Natural competitive inhibitors of ECA	[58]
Barley bran			4540 µg/mL			
Solid-state fermented wheat	Phenolic compounds	in vitro	53.8%	ND	Inhibition of ECA by proteolysis	[59]
Bioprocessed wheat middlings	Phenolic compounds	in vitro	94.9%	ND	The hydrolysis of short chain peptides increases ECA- inhibitory capacity	[60]
Sorghum roasted grain	Phenolic acids and flavonoids	in vitro	20.99 µg/mL	ND	Hydrogen and the hydrophobic union caused by the denaturation of enzymes	[61]
Sorghum grains	Phenolic compounds	in vitro	46.3%	ND	Production of peptides and free amino acids before germination increases ECA-inhibitory activity	[62]
Extruded maize products added with a red seaweed	Phenolic compounds	in vitro	41%	ND	ECA inhibition trough sequestration of enzyme metal factor Zn^2+^	[53]
Water extracts of maize	Soluble phenols	in vitro	50%	ND	Small peptide compounds may represent the bioactive factors contributing to the total ECA-inhibitory activity	[63]

BP: Blood Pressure; SBP: Systolic blood pressure; DBP: Diastolic blood pressure; % IECA: Percent inhibition of ECA; ND: Not determined.

**Table 2:** Peptides derived from cereals with antihypertensive activity.

Food	Bioactive Compound	MW	Test	IC_50_ or % IECA	Decrease BP	Main Mechanism	Reference
Bran of rice	Peptide	<4 kDa	in vitro	30 µg/mL	ND	Reducer and inhibitor of ECA	[67]
Rice protein hydrolysates	Dipeptides	ND	in vitro	76.58-µg/mL	ND	Blocker of ECA due to the presence of aromatic amino acids	[72]
Barley flour	Peptide	<3 kDa	in vitro	70.3%	ND	Inhibitors of ECA via the presence of hydrophobic amino acids	[68]
Corn germ flour	Peptide	<3 kDa	in vivo	830 µg/mL	15.7%	Regulation of vasoconstrictors increases in NO and prostacyclin decreases in Ang II	[73]
Corn germ	Peptides	<6 kDa	in vitro	1389 µg/mL	ND	Inhibitory effect on ECA	[74]
Corn gluten flour	Peptides	<3 kDa	in vivo/in vitro	290 µg/mL	>30 mmHg SBP	Persistent inhibition of the ECA in tissues	[75]
Corn gluten flour	Dipeptide	ND	in vivo-in vitro	37 µg/mL	35–45 mmHg SBP	Inhibitor of ECA by possible synergy between peptides	[76]
Hydrolyzed wheat gluten	Peptides	<1 kDa	in vitro	2 µg/mL	ND	Inhibition of ECA by electrostatic interactions and interactions with hydrogen bonds	[66]
Hydrolyzed wheat gluten	Peptides	<1 kDa	in vitro	4 µg/mL	ND	Competitive and non-competitive inhibitors of ECA	[77]
Defatted wheat germ	Peptides	<5 kDa	in vitro	452 µg/mL	ND	Inhibition of ECA by enzymolysis and ionization of proteins	[78]
Defatted wheat germ	Hydrolyzed proteins	ND	in vitro	220 µg/mL	ND	Inhibition of ECA by hydrophobic amino acids	[79]
Wheat flour	Phenolics from peptide fractions	ND	in vitro	84.52%	ND	Inhibition of ECA by bound phenols after acid hydrolysis	[80]
Oat-isolated protein	Peptides	<3 kDa	in vitro	60%	ND	Ultrasonic pretreated enzymolisis increased ECA-inhibitory activities of the oat peptides	[81]
Oat protein hydrolysate	Peptides	ND	in silico	96.5%	ND	Inhibition of ECA-I by aromatic, small acids with low lipophilicity and high electronic properties	[82]
Oat protein hydrolysate	Peptides	<3 kDa	in vitro e in silico	35 µg/mL	ND	Competitive inhibitors of ECA	[83]
Sweet sorghum grain	Peptides fractions	<1 kDa	in vitro	31.6 µg/mL	ND	Binding of the C-terminal of Serine with the active sites of ECA	[84]
Sorghum protein hydrolysate	Tripeptides	ND	in vitro	1.3 µg/mL	ND	Competitive inhibitor of ECA	[85]
Bread produced with addition of 6% rye-malt gluten	Peptides	ND	in vitro	0.002 µM/mL	ND	ECA binding at the N-terminal and proline or aromatic amino acids at the C-terminus	[86]
Extruded and fermented millet	Peptides	ND	in vivo	ND	14.6%	Reduction in the indexes of RAAS	[87]
Bread or sandwiches with pure millet grains	Protein	ND	Clinical	ND	3%	Inhibition of vasoconstrictors and induction of vasodilators	[88]

BP: Blood Pressure; SBP: Systolic blood pressure; DBP: Diastolic blood pressure; MW: Molecular weight; % IECA: Percent inhibition of ECA; Ala: Alanine; Arg: Arginine; Cys: Cysteine; Gln: Glutamine; Glu: Glutamic acid; Ile: Isoleucine; Leu: Leucine; Lys: Lysine; Phe: Phenylalanine; Pro: Proline; Ser: Serine; Thr: Threonine; Trp: Tryptophan; Tyr: Tyrosine; Val: Valine; NOS: Nitric oxide synthase; ND: Not determined.

## Data Availability

Data is contained within the article.
